# Proportion of patients admitted to rural and remote hospitals that experience a 30-day medication-related readmission: a retrospective cohort study

**DOI:** 10.1007/s11096-026-02228-7

**Published:** 2026-10-07

**Authors:** Kingston Yeung, Gi Kon Nam, Klaudia Englezos, Aagam Bordia, Stephen Carter, Rebekah Moles, Hana Amer, Manya Angley, Charley Budgeon, Brett Chambers, Jenny Chen, Deirdre Criddle, Rohan A. Elliott, Tara Emadi, Cristen Fleming, Deborah Hawthorne, Linda Krogh, Anna Packer, Simon Poon, Frank M. Sanfilippo, Paul Yates, Jerry Yik, Jonathan Penm

**Affiliations:** 1 Faculty of Medicine and Health, School of Pharmacy, The University of Sydney, Sydney, NSW Australia; 2 Department of Pharmacy, The Royal North Shore Hospital, St Leonards, NSW Australia; 3 School of Health and Clinical Sciences, University of Western Australia, Perth, WA Australia; 4 School of Population and Global Health, University of Western Australia, Perth, WA Australia; 5 Cardiovascular Epidemiology Research Centre, University of Western Australia, Perth, WA Australia; 6 Western NSW Local Health District, Dubbo, NSW Australia; 7 Complex Needs Coordination Team and Sir Charles Gairdner Hospital Pharmacy Department, Sir Charles Gairdner, Nedlands, WA Australia; 8 Department of Pharmacy, Austin Health, Heidelberg, VIC Australia; 9 Faculty of Pharmacy and Pharmaceutical Sciences, Monash University, Parkville, VIC Australia; 10 Deborah Hawthorne Consultancy, Wangaratta, VIC Australia; 11 Faculty of Engineering, University of Sydney, Sydney, NSW Australia; 12 Department of Geriatric Medicine, Austin Health, Heidelberg, VIC Australia; 13 Department of Medicine, Austin Health, Heidelberg, VIC Australia; 14 Advanced Pharmacy Australia, Melbourne, VIC Australia; 15 Department of Pharmacy, Prince of Wales Hospital, Randwick, NSW Australia

**Keywords:** Drug-related problems, Drug-related readmissions, Hospital readmission, Medication safety, Risk factors

## Abstract

**Introduction:**

Medication-related readmissions are a significant and preventable cause of patient harm. Patients living in rural and remote areas may face inequitable access to healthcare and fragmented continuity of care, increasing their vulnerability to medication-related harm. Despite this, the burden and drivers of medication-related readmissions in these settings remain poorly understood. Determining the current burden and identifying factors associated with medication-related readmissions is essential to inform targeted strategies to improve medication safety.

**Aim:**

To determine the proportion of adult patients with a medication-related readmission within 30 days of discharge from rural and remote hospitals, and to identify factors potentially associated with these readmissions.

**Method:**

A retrospective cohort study was conducted using medical records of adults admitted to rural and remote hospitals in New South Wales, Australia, between June 1, 2022 and August 31, 2023. Readmissions within 30 days were assessed for medication-related causes using the AT-HARM10, a validated 10-item tool based on admission notes, medication lists, inpatient pathology results, and discharge summaries. Patient demographics, comorbidities and admission details were extracted. Comorbidity burden was calculated using the Charlson Comorbidity Index (CCI). Medication lists were based on medications charted 12 h before discharge. Univariable logistic regression was performed to explore factors associated with 30-day medication-related readmissions, with results reported as unadjusted odds ratios (uOR).

**Results:**

Of the 2220 patients admitted during the study period, 6% (n = 133) experienced a 30-day readmission, of which 22.5% (n = 30) were medication-related. Overall, 1.4% of all admitted patients experienced a 30-day medication-related readmission. Older age (uOR 1.04; 95% CI 1.01–1.07), higher CCI (uOR 1.22; 95% CI 1.04–1.44), a greater number of regular medications (uOR 1.07 per additional medication; 95% CI 1.01–1.14), and insulin use (uOR 2.80; 95% CI 1.06–7.42) were associated with medication-related readmission.

**Conclusion:**

In rural and remote hospitals, a small but clinically important proportion of patients experienced a medication-related readmission within 30 days of discharge. Unadjusted analysis identified that older age, multimorbidity, polypharmacy and insulin use were associated with increased risk. Larger studies are needed to validate these findings and evaluate strategies to reduce medication-related harm in rural and remote populations.

**Supplementary Information:**

The online version contains supplementary material available at https://doi.org/10.1007/s11096-026-02228-7.

## Impact statements

- Almost one in four 30-day readmissions were medication-related, highlighting medication safety as a potential target for reducing avoidable healthcare utilisation in rural and remote populations.
- Medication-related readmissions occurred in 1.4% of all patients admitted to rural and remote hospitals, representing a clinically important burden of medication-related harm.
- Older age, multimorbidity, polypharmacy and insulin use were associated with medication-related readmissions in unadjusted analysis, warranting further investigation in future studies.

## Introduction

Medication-related harm is a significant and preventable cause of patient harm. It may arise from medication errors or from adverse drug reactions that occur despite appropriate medication use [1]. As healthcare becomes increasingly complex, patients are exposed to more medicines and treatment changes, increasing the risk of medication-related harm. Globally, preventable medication-related harm is estimated to affect approximately 5% of patients, with one quarter of these events classified as severe or potentially life-threatening [2]. These events contribute to potentially avoidable morbidity and mortality, prolonged hospital stays, unplanned readmissions and substantial financial healthcare costs [3]. Improving medication safety is an international priority, reflected in the World Health Organisation’s third Global Patient Safety Challenge: Medication Without Harm [4, 5].

Medication-related hospital admissions and readmissions represent an important and potentially preventable consequence of medication-related harm. Previous studies suggest that 65% of medication-related admissions [6] and 69% of medication-related readmissions may be potentially preventable [7]. Transitions of care are recognised as periods of increased vulnerability, as patients move between healthcare settings, and may experience changes to their medication regimens. These changes can increase the risk of medication errors. Approximately half of all adults admitted to the hospital experience a medication error on discharge, with nearly 20% affected by medication-related harm [8]. Common contributing factors include incomplete documentation, poor communication and handover [9], and delays in accessing medicines [10].

These challenges are further amplified in rural and remote communities. These populations often face socioeconomic disadvantages, higher rates of unhealthy lifestyle behaviours [11], and significant challenges in accessing healthcare. Healthcare workforce shortages, difficulties in retaining healthcare workers [12, 13], geographic isolation, and limited transport options [11] all contribute to the complexity of managing these patients. As a result, up to 67% of rural populations are estimated to have limited access to essential health services [14]. Previous studies have reported that, compared to urban populations, rural communities experience significantly higher rates of potentially preventable medication-related readmissions (30% vs. 17%) [15]. Despite these disparities, the burden of medication-related harms in rural and remote populations remains poorly understood, as existing literature has focused mainly on urban or tertiary hospitals [7, 16]. Addressing this evidence gap by determining the proportion of medication-related readmissions in rural and remote hospitals and identifying factors associated with readmission is essential to support clinicians and health systems in identifying patients at greatest risk.

### Aim

The primary objective of this study was to determine the proportion of adult patients discharged from a rural and remote hospital who experienced a medication-related readmission within 30 days. The secondary objective was to identify potential factors associated with these 30-day medication-related readmissions.

## Method

This study was reported in accordance with the Strengthening the Reporting of Observational Studies in Epidemiology (STROBE) checklist for cohort studies [17].

### Study design and setting

This retrospective cohort study examined patient data extracted from digital hospital medical records over a 15-month period.

Participants were admitted to 19 rural and remote hospitals within the Western New South Wales Local Health District (WNSWLHD). The WNSWLHD covers more than 250,000 square kilometres and serves approximately 309,700 people [18]. The district reports 15% higher rates of preventable hospitalisations compared with the rest of NSW [18]. The WNSWLHD serves a mixture of rural and remote communities, with participating hospitals ranging in size from 2 to 25 beds. These hospitals were located between 50 and 200 km from the regional centre [18].

The Modified Monash Model (MMM) categorises areas by geographical remoteness and population size, ranging from MM1 (metropolitan areas) to MM7 (very remote communities) [19]. All participating hospitals were located in areas classified as MM4 (medium rural towns) to MM6 (remote communities).

### Participants

Eligible participants were adults aged ≥18 years who had their index hospital admission between 1 June 2022 and 31 August 2023. Patients were excluded if they presented only to the emergency department (without inpatient admission) or attended an outpatient clinic.

### Outcomes

The primary outcome was whether the patient had a medication-related readmission within 30 days of discharge. All readmissions, including elective and planned readmissions, were assessed using the AT-HARM10 tool [20, 21]. The AT-HARM10 is a validated 10-item tool that requires “Yes” or “No” responses based on a review of admission notes, medication lists, inpatient pathology results, and discharge summaries. Questions are answered systematically until a “Yes” is reached. Questions 1 to 3 identify admissions unlikely to be medication-related, then questions 4 to 10 assess the likelihood that the admission had a medication-related cause. A “Yes” to any of questions 1 to 3 classifies the admission as unlikely medication-related, while a “Yes” to any of questions 4 to 10 indicates a medication-related readmission.

All readmission cases were independently reviewed by at least two reviewers: KE (registered pharmacist) and GN (final-year pharmacy student). To ensure consistency and reliability, 20% of cases were randomly selected for review by a third reviewer, KY (registered pharmacist). KY adjudicated any discrepancies to reach a final decision.

### Data collection

Patient demographics were selected based on previous literature examining predictors of hospital readmission [22]. These included age, gender, Aboriginal or Torres Strait Islander status, whether an interpreter was required, postcode, ICD-10-AM codes, and details related to the index admission. Data were extracted from electronic medical records (across the 19 participating sites. All sites used the same electronic medical record, PowerChart (Oracle Cerner, Redwood Shores, California, United States).

To ensure data reliability, an independent pharmacist researcher (KY) independently reviewed 5% of cases during the data extraction process. Variables related to the index admission included the length of stay and medications charted during admission. Due to data availability constraints, medication data were limited to those charted 12 h prior to discharge. Medications prescribed on an as-needed or “PRN” basis were excluded.

Comorbidities were identified, and the Charlson Comorbidity Index (CCI) was calculated using ICD-10-AM codes recorded in the digital records (Supplementary Data 1). The CCI is a validated scoring of a patient’s comorbidity burden, used to estimate mortality risk based on the number and severity of comorbidities [23]. A higher CCI score indicates a higher comorbidity burden and an associated increase in predicted mortality risk.

Patient postcodes were used to classify the participants’ residential location according to the MMM. Due to the distribution of participants across MMM categories, MMM classification was analysed as a binary variable in the univariate logistic regression, comprising non-remote (MM1-5) and remote (MM6-7) areas.

### Statistical analysis

Descriptive statistics were used to summarise patient demographics. Continuous variables that were normally distributed were reported using mean (Standard Deviation), and non-normally distributed variables were reported as median (interquartile range (Q1-Q3)). Potential predictors of medication-related readmission were selected through a combination of existing readmission literature [7, 16, 24, 25] and the clinical expertise of the research team. Comorbidities were selected using the Australian Institute of Health and Welfare (AIHW) list of common chronic conditions [26]. These included cancer; chronic musculoskeletal conditions; chronic respiratory conditions; dementia; diabetes; heart disease, stroke and vascular disease; and mental health disorders. Additional comorbidities frequently associated with readmission, including neoplasm, heart failure, chronic kidney disease, ischaemic heart disease, liver disease and peptic ulcer disease, were also included [24, 25].

Medication classes were selected based on previous Australian high-risk medication lists [27]. These included antibiotics, insulin, opioids and benzodiazepines, chemotherapy, heparins (including low-molecular-weight heparins (LMWH) and anticoagulants. Additional medication classes associated with readmission, including psychotropics, were also included [7, 16, 24]. Psychotropics were defined as antidepressants, antiepileptics or antiparkinsonian medications [24]. Medicines within each class were identified using the Anatomical Therapeutic Chemical (ATC) classification system (Supplementary Data 2) [28].

Each variable was analysed separately using univariable logistic regression to identify potential factors associated with 30-day medication-related readmissions, comparing patients who had a medication-related readmission with those who did not have a readmission. Given the small number of events (n = 30) relative to the number of variables explored, multivariate modelling was not undertaken due to the substantial risk of overfitting and model instability. Therefore, all reported odds ratios are unadjusted (uOR). Analysis was completed using IBM SPSS Statistics (version 26; IBM Corp, Armonk, New York, United States).

As this was an exploratory study, no formal sample size was pre-determined. Non-medication-related readmissions were excluded from the analysis.

### Ethics approval

All research materials and protocols were approved by the Western New South Wales Local Health District Ethics Committee (HREC: 2023/ETH01292).

### Results

#### Participant characteristics

In total, 2220 adult patients were admitted to rural and remote hospitals in the catchment area during the study period. Patient demographics are summarised in Table 1.

**Table 1:** Characteristics of admitted patients and univariable logistic regression of covariates associated with 30-day medication-related readmission

Variable	Total cohort (n=2220)	30-day medication-related readmission (n=30)	No 30-day medication-related readmission (n=2190)	Unadjusted OR (95% CI)
Demographic				
Sex; female	1161 (52.3%)	16 (53.3%)	1145 (52.3%)	1.04 (0.51–2.15)
Identifies as Aboriginal or Torres Strait Islander	268 (12.1%)	4 (13.3%)	264 (12.1%)	1.12 (0.39–3.24)
Age; years	72.4 ± 15.9	79.4 ± 10.2	72.3 ± 16.0	1.04 (1.01–1.07)
Interpreter required	2 (0.1%)	0 (0.0%)	2 (0.1%)	NA
Rurality				
Lives non-remote	1958 (88.2%)	24 (80.0%)	1934 (88.3%)	Reference
Lives remote	262 (11.8%)	6 (20.0%)	256 (11.7%)	1.89 (0.77–4.66)
Number of comorbidities	5 [3–7]	6 [3–7]	5 [3–7]	1.17 (0.99–1.38)
CCI score	3.4 ± 1.8	4.2 ± 1.3	3.4 ± 1.9	1.22 (1.04–1.44)
Comorbidities				
Cancer	66 (3.0%)	0 (0.0%)	66 (3.0%)	NA
Chronic musculoskeletal conditions	76 (3.4%)	0 (0.0%)	76 (3.5%)	NA
Chronic respiratory conditions	132 (5.9%)	3 (10.0%)	129 (5.9%)	1.78 (0.53–5.93)
Dementia	97 (4.4%)	2 (6.7%)	95 (4.3%)	1.58 (0.37–6.71)
Diabetes	502 (22.6%)	8 (26.7%)	494 (22.6%)	1.25 (0.55–2.82)
Heart, stroke and vascular disease	363 (16.4%)	7 (23.3%)	356 (16.3%)	1.57 (0.67–3.68)
Mental Health Disorder	201 (9.1%)	1 (3.3%)	200 (9.1%)	0.34 (0.05–2.53)
Heart Failure	78 (3.5%)	1 (3.3%)	77 (3.5%)	0.95 (0.13–7.04)
Chronic Kidney Disease	224 (10.1%)	4 (13.3%)	220 (10.0%)	1.38 (0.48–3.98)
Ischaemic Heart Disease	30 (1.4%)	0 (0.0%)	30 (1.4%)	NA
Liver disease	15 (0.7%)	1 (3.3%)	14 (0.6%)	5.36 (0.68–42.12)
Peptic Ulcer Disease	0 (0.0%)	0 (0.0%)	0 (0.0%)	NA
Medicines				
Number of medicines	5 [2–9]	8.5 [2–12]	5 [2–9]	1.07 (1.01–1.14)
Antibiotics	755 (34.0%)	13 (43.3%)	742 (33.9%)	1.49 (0.72–3.09)
Insulin	151 (6.8%)	5 (16.7%)	146 (6.7%)	2.80 (1.06–7.42)
Opioids or Benzodiazepines	379 (17.1%)	9 (30.0%)	370 (16.9%)	2.11 (0.96–4.64)
Chemotherapy	39 (1.8%)	1 (3.3%)	38 (1.7%)	1.95 (0.26–14.71)
Heparin or anticoagulants	956 (43.1%)	12 (40.0%)	944 (43.1%)	0.88 (0.42–1.84)
Psychotropics	474 (21.4%)	5 (16.7%)	469 (21.4%)	0.73 (0.28–1.93)
Index admission				
Length of stay	4 [3–8]	4.5 [3–7.8]	4 [3–8]	1.00 (0.96–1.03)

Data is presented as n (%), mean ± standard deviation (SD), or median [interquartile range (Q1-Q3)]; CCI, Charlson Comorbidity Index; NA, Not applicable as one group had no cases

Overall, 52.3% (n = 1161) of patients were female, and the mean age was 72.4 years (SD 15.9). The majority of patients resided in non-remote areas (MM 1–5, n = 1958, 88.2%), with 12.1% (n = 268) identifying as Aboriginal or Torres Strait Islander.

Patients had a median of 5 comorbidities (Q1–Q3 3–7) and a mean CCI score of 3.4 (SD 1.8). The most common comorbidities were diabetes (n = 502, 22.6%); heart disease, stroke or vascular disease (n = 363, 16.4%); chronic kidney disease (n = 224, 10.1%); and mental health disorders (n = 201, 9.1%).

During their index admission, the median length of stay was four days (Q1–Q3 3–8). Twelve hours prior to discharge, patients had a median of five regular medicines charted (Q1–Q3 2–9). The most frequently prescribed high-risk medicines were heparins (including LMWH) and anticoagulants (n = 956 patients, 43.1%), antibiotics (n = 755 patients, 34.0%), and psychotropics (n = 474 patients, 21.4%).

#### Proportion of patients experiencing a 30-day medication-related readmission

Of the 2220 patients admitted, 133 (6.0%) were readmitted within 30 days. Of these, 30 (22.5%) were identified as medication-related (Table 2). Of the 2220 patients, 1.4% (n = 30) were identified as experiencing a 30-day medication-related readmission.

**Table 2:** Classification of 30-day medication-related readmissions using the AT-HARM 10 tool

30-day readmissions during the study period	n = 133
Readmission due to infection, previously undiagnosed disease (e.g., diabetes or heart failure), or symptoms, signs and abnormal clinical and laboratory findings (if no specific diagnosis has been made) that do not seem to be drug-related	n = 48 (36.1%)
Readmission caused by progression of a previously diagnosed disease that is not drug-related	n = 37 (27.8%)
Admission caused by physical trauma, substance intoxication, social circumstances or allergies (e.g., car accident, alcohol, mushroom poisoning, wasp allergy) that was/were not drug-related	n = 18 (13.5%)
Potentially medication-related readmission	n = 30 (22.6%)

#### Univariable factors associated with a 30-day medication-related readmission

The univariable logistic regression model identified several variables associated with a 30-day medication-related readmission (Table 1). Older age (uOR 1.04; 95% CI 1.01–1.07), higher CCI score (uOR 1.22; 95% CI 1.04–1.44), greater number of regular medicines (uOR 1.07 per additional medication; 95% CI 1.01–1.14), and the prescription of insulin (uOR 2.80; 95% CI 1.06–7.42) were each associated with increased odds of medication-related readmission.

## Discussion

This cohort study found that 1.4% of patients admitted to rural and remote hospitals experienced a 30-day medication-related readmission. This estimate is consistent with a previous systematic review reporting medication-related readmission rates ranging from 0.3 to 18% among hospitalised patients [7]. The wide variation observed across studies likely reflects differences in study design, patient populations, healthcare settings, and follow-up durations. In the systematic review, follow-up periods ranged from 28 days to 4.2 years, with only seven reported outcomes within 30 days. Of these, only two provided overall prevalence estimates, which ranged from 2 to 4.3% [29, 30]. Moreover, most studies were conducted in metropolitan teaching hospitals, whereas this cohort focused on smaller rural and remote facilities. Despite these differences in setting, the proportion of medication-related 30-day readmissions observed in this cohort was comparable to rates reported in urban tertiary hospitals.

Beyond overall prevalence, this study identified several factors associated with an increased unadjusted risk of 30-day medication-related readmission, including older age, higher CCI, greater medication use and insulin use. Older patients and polypharmacy have been consistently reported in previous systematic reviews as associated with medication-related readmissions [16, 24, 31]. Rather than individual comorbidities, the CCI score was associated with the risk of medication-related readmissions in this cohort. Although originally developed to predict mortality, the CCI reflects cumulative disease burden, which may better capture the complexity that contributes to medication-related harm [32]. Previous reviews have identified specific conditions, such as cancer, heart failure, chronic kidney disease, ischaemic heart disease, liver disease and peptic ulcer disease, as associated with medication-related readmission [24, 25]. While these individual conditions were not independently associated with medication-related readmission in this study, they are incorporated within the CCI scoring system. This suggests that overall multimorbidity, rather than specific comorbidities, may be more clinically meaningful when identifying patients at higher risk. Targeting interventions towards older patients with substantial comorbidity burden and polypharmacy may therefore help reduce medication-related harm; however, further studies are needed to validate these findings.

High-risk medication classes are frequently used to guide clinical prioritisation. In this study, insulin prescription was associated with an increased unadjusted risk of medication-related readmission. Insulin is recognised as a high-risk medicine due to its narrow therapeutic window and the potential consequences of both underdosing and overdosing. Inadequate dosing can result in poor glycaemic control and diabetes-related complications [33], while excessive dosing increases the risk of hypoglycaemia [34]. These findings are consistent with previous literature. A cross-sectional study conducted in a tertiary teaching hospital reported that 10% of preventable medication-related readmissions were attributed to insulin [35]. Similarly, another cohort study in rural and remote hospitals identified insulin as a causal medication in 5% of medication-related readmissions, with overuse contributing to hypoglycaemia-related falls and underuse leading to diabetic ketoacidosis [36]. These risks may be amplified in rural and remote areas, where logistical barriers, costs, transport limitations and reduced access to healthcare services can limit effective diabetes management [37]. These barriers can disrupt timely medication supply and reduce access to diabetes support, including insulin titration, monitoring and early intervention when glucose levels become unstable. Given these challenges, patients prescribed insulin may require additional support during discharge planning. Strategies such as ensuring continuity of medication supply and providing tailored education may help reduce medication-related harm and the risk of readmission.

This study had several strengths. Inclusion of patients across a range of medical specialties enhanced the generalisability of the findings across diverse clinical areas. Variables included in the univariable logistic regression were informed by the research team’s clinical expertise and supported by existing literature, ensuring analysis was grounded in both clinical experience and evidence [16, 24].

However, several limitations should be considered when interpreting these results. First, the AT-HARM10 tool [20, 21] was initially developed and validated primarily in older patients, whereas this study included all adults ≥18 years. Although the tool contains no age-specific criteria, and no difficulties were encountered when applying it to younger adults during outcome assessment, its performance in younger adult populations has not been formally validated.

Second, the analysis was limited to univariable models. With only 30 medication-related readmission events, multivariable modelling was not feasible due to the high risk of overfitting and unstable estimates. As a result, the reported odds ratios are unadjusted and may be confounded by or subject to unidentified interactions among confounders. The identified associations should therefore be considered exploratory and hypothesis-generating, rather than evidence of independent risk factors. Validation in larger cohorts with sufficient events to support robust multivariable analysis is required.

Third, all readmissions, including planned or elective readmissions, were assessed for medication-relatedness to ensure consistent outcome assessment using the AT-HARM10 tool. However, as planned admissions may reflect different clinical pathways than unplanned readmissions, comparisons with studies that exclude planned admissions should be interpreted with caution.

Lastly, as a retrospective observational study with unmeasured confounders, causality cannot be established between identified factors and medication-related readmissions. The study also relied on clinician documentation, which may be incomplete or inconsistent, introducing potential bias. Medication data were extracted 12 h before discharge to standardise data collection, as discharge medication lists were inconsistently documented and discharge medication errors are common [8]. However, this may not accurately reflect patients’ discharge regimens, potentially leading to over- or underestimation of high-risk medication use. Future studies should incorporate prospective data collection or linked health datasets and utilise reconciled discharge medication lists to assess the relationship between high-risk medications and medication-related readmissions more accurately. Finally, the preventability of medication-related readmissions was not assessed. Previous studies suggest that between 15 and 22% of medication-related readmissions in older patients may be preventable [24]. Further studies are needed to evaluate the preventability of these events and the effectiveness of targeted interventions to reduce risk among high-risk patients.

## Conclusion

This study found that a small but clinically important proportion of patients (1.4%) admitted to rural and remote hospitals experienced a medication-related readmission within 30 days. In unadjusted univariate analysis, older age, multimorbidity, polypharmacy and insulin use were associated with an increased risk of medication-related readmission. These findings highlight the importance of identifying patients at higher risk of medication-related harm and providing targeted medication safety interventions. This is particularly important in rural and remote settings, where barriers to healthcare access may increase vulnerability to medication-related harm. Further studies using larger cohorts are required to validate these findings and evaluate strategies to reduce medication-related harm in rural and remote populations.

## Supplementary Information

Below is the link to the electronic supplementary material.

Supplementary file1 (DOCX 23 kb)

## Data Availability

The data used in this study was obtained from the Western New South Wales Local Health District for the purpose of this study. Access to these data is restricted and not publicly available. However, de-identified data may be made available upon reasonable request.
