# Supplementary material for: Proportion of patients admitted to rural and remote hospitals that experience a 30-day medication-related readmission: a retrospective cohort study
Source: Int J Clin Pharm. 2026 Oct 7;48(6):2504–12. doi: 10.1007/s11096-026-02228-7 (PMC13731506; doi:10.1007/s11096-026-02228-7)
Supplement: Supplementary file 1 [file 11096_2026_2228_MOESM1_ESM.docx]

**List of Supplementary Data**

**Title:** Proportion of patients admitted to rural and remote hospitals that experience a 30-day medication-related readmission: a retrospective cohort study

**Journal:** International Journal of Clinical Pharmacy (IJCP)

**Authors:** *Kingston Yeung,^1^ Gi Kon Nam,^1^ Klaudia Englezos,^1^ Aagam Bordia,^2^ Stephen Carter,^1^ Rebekah Moles,^1^ Hana Amer,^1^ Manya Angley,^3^ Charley Budgeon,^4,5^ Brett Chambers,^6^ Jenny Chen,^1^ Deirdre Criddle,^7^ Rohan A Elliott,^8,9^ Tara Emadi,^1^ Cristen Fleming,^6^ Deborah Hawthorne,^10^ Linda Krogh,^6^ Anna Packer,^6^ Simon Poon,^11^ Frank M Sanfilippo,^4,5^ Paul Yates,^12, 13^ Jerry Yik^14^ and Jonathan Penm^1, 15^

**Authors’ Affiliations:**

*^1^Faculty of Medicine and Health, The University of Sydney, Sydney NSW, Australia*

*^2^Department of Pharmacy, The Royal North Shore Hospital, St Leonards NSW, Australia*

*^3^School of Health and Clinical Sciences, University of Western Australia, Perth WA, Australia*

*^4^School of Population and Global Health, University of Western Australia, Perth WA, Australia*

*^5^Cardiovascular Epidemiology Research Centre, University of Western Australia, Perth WA, Australia*

*^6^Western NSW Local Health District NSW, Australia*

*^7^Complex Needs Coordination Team and Sir Charles Gairdner Hospital Pharmacy Department, Sir Charles Gairdner, Nedlands WA, Australia*

*^8^Department of Pharmacy, Austin Health, Heidelberg VIC, Australia*

*^9^Faculty of Pharmacy and Pharmaceutical Sciences, Monash University, Parkville VIC, Australia*

*^10^Deborah Hawthorne Consultancy, VIC, Australia*

*^11^Faculty of Engineering, University of Sydney, Sydney NSW, Australia*

*^12^Department of Geriatric Medicine, Austin Health, Heidelberg VIC, Australia*

*^13^Department of Medicine, Austin Health, Heidelberg VIC, Australia*

*^14^Advanced Pharmacy Australia, Melbourne VIC, Australia*

*^15^Department of Pharmacy, Prince of Wales Hospital, Randwick NSW, Australia*

*Correspondence should be addressed to Kingston Yeung

Address: School of Pharmacy, Faculty of Medicine and Health, The University of Sydney

Email: [kingston.yeung@sydney.edu.au](mailto:kingston.yeung@sydney.edu.au)

**Supplementary Data 1: ICD-10-AM Disease Codes**

| **Comorbidity** | **ICD-10-AM Disease Codes** |
| --- | --- |
| Cancer | C000–C006, C008–C010, C020–C024, C028–C031, C039–C041, C048–C052, C058–C062, C068–C069, C07, C080–C081, C088–C089, C090–C091, C098–C099, C100–C104, C108–C109, C110–C113, C118–C119, C12, C130–C132, C138–C139, C140, C142, C148, C150–C155, C158–C159, C160–C166, C168–C169, C170–C173, C178–C179, C180–C189, C19, C20, C210–C212, C218, C220–C224, C227, C229, C23, C240–C241, C248–C249, C250–C254, C257–C259, C260–C261, C268–C269, C300–C301, C310–C313, C318–C319, C320–C323, C328–C329, C33, C340–C343, C348–C349, C37, C380–C384, C388, C390, C398–C399, C400–C403, C408–C409, C4101–C4102, C411–C414, C418–C419, C430–C439, C440–C449, C450–C452, C457, C459, C460–C463, C467–C469, C470–C476, C478–C479, C480–C482, C488, C490–C496, C498–C499, C500–C506, C508–C509, C510–C512, C518–C519, C52, C530–C543, C548–C549, C55, C56, C570–C574, C577–C579, C58, C600–C602, C608–C609, C61, C620–C621, C629, C630–C632, C637–C639, C64, C65, C66, C670–C679, C680–C681, C688–C689, C690–C699, C700–C701, C709, C710–C719, C720–C725, C728–C729, C73, C740–C741, C749, C750–C755, C758–C759, C760–C762, C7631–C7632, C7639, C764–C765, C767–C768, C770–C775, C778–C779, C780–C788, C790–C797, C7981, C7983–C7984, C7988, C799, C800, C809, C810–C814, C817, C819, C820–C827, C829, C830–C831, C833, C835, C837–C839, C840–C841, C844–C849, C851–C852, C857, C859, C860–C866, C8800–C8801, C8820–C8821, C8830–C8831, C8840–C8841, C8870–C8871, C8890–C8891, C9000–C9001, C9010–C9011, C9020–C9021, C9030–C9031, C9100–C9101, C9110–C9111, C9130–C9131, C9140–C9141, C9150–C9151, C9160–C9161, C9170–C9171, C9180–C9181, C9190–C9191, C9200–C9201, C9210–C9211, C9220–C9221, C9230–C9231, C9240–C9241, C9250–C9251, C9260–C9261, C9270–C9271, C9280–C9281, C9290–C9291, C9300–C9301, C9310–C9311, C9330–C9331, C9370–C9371, C9390–C9391, C9400–C9401, C9420–C9421, C9430–C9431, C9440–C9441, C9460–C9461, C9470–C9471, C9500–C9501, C9510–C9511, C9570–C9571, C9590–C9591, C960, C962, C964–C969, D000–D002, D010–D015, D017, D019, D020–D024, D030–D039, D040–D049, D050–D051, D057, D059, D060–D061, D067, D069, D070–D076, D090–D093, D097, D099, D100–D107, D109–D110, D117, D119, D120–D129, D130–D137, D139, D140–D144, D150–D152, D157, D159, D160–D163, D1641–D1642, D165–D169, D170–D177, D179, D1800–D1808, D181, D190–D191, D197, D199, D200–D201, D210–D216, D219, D220–D227, D229, D230–D237, D239, D24, D250–D252, D259, D260–D261, D267, D269, D27, D280–D282, D287, D289, D290–D294, D297, D299, D300–D304, D307, D309, D310–D317, D319, D320–D321, D329, D330–D334, D337, D339, D34, D350–D356, D357–D359, D360–D361, D367, D369, D370–D376, D3771, D3779, D379–D386, D390–D392, D397, D399, D400–D401, D407, D409, D410–D414, D417, D419, D420–D421, D429, D430–D434, D437, D439, D440–D449, D45, D460–D466, D467, D469, D470–D475, D477, D479, D480–D487, D489 |
| Chronic Kidney Disease | E1021–E1022, E1029, E1121–E1122, E1129, E1321–E1322, E1329, E1421–E1422, E1429, I120, I130–I132, I139, I151, N000–N009, N010–N019, N020–N029, N030–N039, N040–N049, N050–N059, N060–N069, N070–N079, N080–N082, N084–N085, N088, N111, N118–N119, N12, N140–N144, N150–N151, N158–N159, N160–N165, N168, N181–N185, N189, N19, N250–N251, N258–N259, N26, N270–N271, N279, N280–N281, N288–N289, N391–N392, D593, B520, E853, Q600–Q606, Q610–Q6145, Q6150–Q6152, Q618–Q620, Q6215–Q6217, Q6219, Q622, Q6230–Q6235, Q6239, Q624, Q6251–Q6252, Q6259, Q6260–Q6266, Q6269, Q6270–Q6272, Q628, Q6301–Q6302, Q6309, Q6310–Q6312, Q6319, Q6320–Q6323, Q6329, Q633, Q6381–Q6382, Q6389, Q639, T824, T861, Z490, Z940, Z992, U871 |
| Chronic Musculoskeletal Conditions | M0230–M0239, M0210–M0219, M0220–M0229, M0230–M0239, M0500–M0509, M0510–M0519, M0520–M0529, M0530–M0539, M0580–M0589, M0590–M0599, M0600–M0609, M0610–M0619, M0620–M0629, M0630–M0639, M0640–M0649, M0680–M0689, M0690–M0699, M0800–M0809, M0810–M0819, M0820–M0829, M083, M0840–M0849, M0880–M0889, M1000–M1009, M1010–M1019, M1030–M1039, M1040–M1049, M1090–M1099, M1280–M1289, M130, M1311–M1319, M1380–M1389, M1390–M1399, M150–M154, M158–M159, M160–M161, M162–M167, M169, M170–M175, M179, M180–M181, M182–M185, M189, M1901–M1904, M1907–M1909, M1911–M1914, M1917–M1919, M1921–M1924, M1927–M1929, M1981–M1984, M1987–M1989, M1991–M1994, M1997–M1999, M4000–M4009, M4010–M4019, M4020–M4029, M4030, M4035–M4039, M4040–M4049, M4050–M4059, M4100–M4109, M4110–M4119, M4120–M4129, M4130–M4139, M4140–M4149, M4150–M4159, M4180–M4189, M4190–M4199, M4200–M4209, M4210–M4219, M4290–M4299, M4300–M4309, M4310–M4319, M4320–M4329, M433, M434, M4350, M4352–M4359, M436, M4380–M4389, M4390–M4399, M4500–M4509, M4600–M4609, M461, M4620–M4629, M4630–M4639, M4640–M4649, M4650–M4659, M4680–M4689, M4690–M4699, M4700–M4709, M4710–M4719, M4720–M4729, M4780–M4789, M4790–M4799, M4800–M4809, M4810–M4819, M4820–M4829, M4830–M4839, M4840–M4849, M4850–M4859, M4880–M4889, M4890–M4899, M4900–M4909, M4910–M4919, M4920–M4929, M4930–M4939, M4940–M4949, M4950–M4959, M4980–M4989, M500–M503, M508–M509, M510–M514, M518–M519, M530–M531, M5320–M5329, M533, M5380–M5389, M5390–M5399, M5400–M5409, M5410–M5419, M542–M546, M5480–M5489, M5490–M5499, M990–M999, M8000–M8009, M8010–M8019, M8020–M8029, M8030–M8039, M8040–M8049, M8050–M8059, M8080–M8089, M8090–M8099, M8100–M8109, M8110–M8119, M8120–M8129, M8130–M8139, M8140–M8149, M8150–M8159, M8160–M8169, M8180–M8189, M8190–M8199, M8200–M8209, M8210–M8219, M8280–M8289 |
| Chronic Respiratory Conditions | J40, J410–J412, J418, J42, J430–J432, J438–J439, J440–J441, J448–J449, J450–J451, J458–J459, R05, R060–R062, J40, J410–J412, J418, J42, J430–J432, J438–J439, J440–J441, J448–J449 |
| Dementia | F0000–F0001, F0020, F0090, F0100–F0101, F0110, F0120, F0130, F0180, F0190, F0200, F0210, F0220, F0230, F0240, F0280, F0300, F051, F107, F1370–F1371, F1379, F187, G300–G301, G308–G309, G311–G313, G318–G319, U791 |
| Diabetes | E0921, E0929, E0931–E0932, E0940, E0942, E0951–E0952, E0971–E0972, E098–E099, E1001–E1002, E1011–E1016, E1021–E1022, E1029, E1031–E1036, E1039, E1040–E1043, E1049, E1051–E1053, E1061–E1065, E1069, E1071, E1073, E108–E109, E1101–E1102, E1111–E1116, E1121–E1122, E1129, E1131–E1136, E1139, E1140–E1143, E1149, E1151–E1153, E1161–E1165, E1169, E1171–E1173, E118–E119, E1301–E1302, E1311–E1316, E1321–E1322, E1329, E1331–E1336, E1339, E1340–E1343, E1349, E1351–E1353, E1361–E1365, E1369, E1371–E1373, E138–E139 |
| Heart Failure | I110, I130, I132, I500–I501, I509 |
| Heart, Stroke and Vascular Disease | I00, I010–I012, I018–I019, I020, I029, I050–I052, I058–I059, I060–I062, I068–I069, I070–I072, I078–I079, I080–I083, I088–I089, I090–I092, I098–I099, I10, I110, I119, I120, I129, I130–I132, I139, I150–I152, I158–I159, I200–I201, I208–I209, I210–I214, I219, I220–I221, I228–I229, I230–I236, I238, I240–I241, I248–I249, I250, I2510–I2513, I252–I256, I258–I259, I260, I269, I270–I272, I278–I279, I280–I281, I288–I289, I300–I301, I308–I309, I310–I313, I318–I319, I320–I321, I328, I330, I339, I340–I342, I348–I349, I350–I352, I358–I359, I360–I362, I368–I369, I370–I372, I378–I379, I38, I390–I394, I398, I400–I401, I408–I409, I410–I412, I418, I420–I429, I430–I432, I438, I440–I447, I450–I456, I458–I459, I460–I461, I469, I470–I472, I479, I480–I484, I489, I490–I4951, I498–I499, I500–I501, I509, I510–I519, I520–I521, I528, I600–I609, I610–I616, I618–I619, I620–I621, I629, I630–I636, I638–I639, I64, I650–I653, I658–I659, I660–I664, I668–I669, I670–I679, I680–I682, I688, I690–I694, I698, I700–I7024, I708–I709, I7100–I7103, I711–I716, I718–I719, I720–I725, I726, I728–I729, I7300–I7301, I731, I738–I739, I740–I745, I748–I749, I770–I776, I778–I779, I780–I781, I788–I789, I790–I792, I798, I800–I801, I8020–I8023, I803, I8040–I8042, I808–I809, I81, I820–I823, I828–I829, I830–I832, I839, I850, I859, I860–I865, I868, I870–I872, I878–I879, I880–I881, I888–I889, I890–I891, I898–I899, I950, I9510–I9512, I9519, I952, I958–I959, I970, I972, I9731–I9735, I9739, I9781–I9783, I9789, I979, I980–I983, I988, I99, G450–G454, G458–G459, Q200–Q202, Q2030–Q2031, Q2039, Q2040–Q2042, Q2049, Q2050–Q2052, Q2059, Q206, Q2081–Q2082, Q2089, Q209, Q2100–Q2104, Q2109, Q2110–Q2115, Q2119, Q2120–Q2124, Q2129, Q213–Q214, Q2181–Q2183, Q2189, Q219–Q222, Q2230–Q2231, Q2239, Q2241–Q2242, Q225–Q226, Q2281–Q2282, Q2289, Q229, Q2301–Q2302, Q231, Q2321–Q2322, Q233–Q234, Q2381–Q2383, Q2389, Q239–Q246, Q2481–Q2487, Q2489, Q249, Q250, Q2510–Q2513, Q2519, Q252, Q2530–Q2532, Q2539, Q2540–Q2547, Q2549, Q255–Q256, Q2570–Q2571, Q2579, Q258–Q259, Q2600–Q2602, Q2609, Q261–Q266, Q2681, Q2689, Q269 |
| Ischaemic Heart Disease | I200–I201, I208–I209, I210–I214, I219, I220–I221, I228–I229, I230–I236, I238, I240–I241, I248–I249, I250, I2510–I2513, I252–I256, I258–I259 |
| Liver Disease | K700–K704, K709, K710–K719, K720–K721, K729, K730–K732, K738–K739, K740–K746, K750–K754, K758–K759, K760–K769, K770, K778 |
| Mental Health Disorders | F0000–F0001, F0010–F0011, F0020–F0021, F0090–F0091, F0100–F0101, F0110–F0111, F0120–F0121, F0130–F0131, F0180–F0181, F0190–F0191, F0200–F0201, F0210–F0211, F0220–F0221, F0230–F0231, F0240–F0241, F0280–F0281, F0300–F0301, F0400–F0403, F049, F050–F051, F058–F059, F060–F062, F0630–F0634, F0639, F064–F069, F070–F072, F078–F079, F09, F100–F109, F110–F119, F120–F129, F1300–F1301, F1309, F1310–F1311, F1319, F1320–F1321, F1329, F1330–F1331, F1339, F1340–F1341, F1349, F1350–F1351, F1359, F1360–F1361, F1369, F1370–F1371, F1379, F1380–F1381, F1389, F1390–F1391, F1399, F140–F149, F1500–F1502, F1509, F1510–F1512, F1519, F1520–F1522, F1529, F1530–F1532, F1539, F1540–F1542, F1549, F1550–F1552, F1559, F1560–F1562, F1569, F1570–F1572, F1579, F1580–F1582, F1589, F1590–F1592, F1599, F1600–F1601, F1609, F1610–F1611, F1619, F1620–F1621, F1629, F1630–F1631, F1639, F1640–F1641, F1649, F1650–F1651, F1659, F1660–F1661, F1669, F1670–F1671, F1679, F1680–F1681, F1689, F1690–F1691, F1699, F170–F179, F180–F189, F190–F199, F200–F206, F208–F209, F21, F220, F228–F229, F2300–F2301, F2310–F2311, F2320–F2321, F2330–F2331, F2380–F2381, F2390–F2391, F24, F250–F252, F258–F259, F28–F29, F300–F302, F308–F309, F310–F317, F318–F319, F3200–F3201, F3210–F3211, F3220–F3221, F3230–F3231, F3280–F3281, F3290–F3291, F330–F334, F338–F339, F340–F341, F348–F349, F380–F381, F388, F39, F4000–F4001, F401–F402, F408–F409, F410–F413, F418–F419, F420–F422, F428–F429, F430–F432, F438–F439, F440–F447, F4480–F4482, F4488, F449, F450–F452, F4530–F4535, F4538–F4539, F454, F458–F459, F480–F481, F488–F489, F500–F505, F508–F509, F510–F515, F518–F519, F520–F529, F530–F531, F538–F539, F54, F550–F556, F558–F559, F59, F600–F602, F6030–F6031, F604–F609, F61, F620–F621, F628–F629, F630–F633, F638–F639, F64, F650–F656, F658–F659, F66, F680–F681, F688, F69, F700–F701, F708–F709, F710–F711, F718–F719, F720–F721, F728–F729, F730–F731, F738–F739, F780–F781, F788–F789, F790–F791, F798–F799, F800–F803, F808–F809, F810–F813, F818–F819, F82–F84, F88–F89, F900–F901, F908–F909, F910–F913, F918–F919, F920, F928–F929, F930–F933, F938–F939, F940–F942, F948–F949, F950–F952, F958–F959, F980–F986, F988–F989, F99, G300–G301, G308–G309, G470–G472, G478–G479, O9931, R440–R443, R448, R450–R451, R454, R480–R482, R488, Z004, Z032, Z046, Z093, Z133, Z502–Z503, Z543, Z619, Z631, Z638–Z639, Z658–Z659, Z714–Z715, Z760 |
| Peptic Ulcer Disease | K270–K277, K279 |

**Supplementary Data 2: High-risk medications**

| **High-risk medications** | ATC Drug codes | ATC Description |
| --- | --- | --- |
| Antibiotics | J01 | Antibacterials for systemic use |
| Insulin | A10A | Insulins and analogues |
| Opioids and benzodiazepines | N02A | Opioids |
|  | N03AE | Benzodiazepine derivatives |
|  | N05BA | Benzodiazepine derivatives |
|  | N05CD | Benzodiazepine derivatives |
| Chemotherapy | L01 | Antineoplastic agents |
| Heparin (including low-molecular-weight heparins) and anticoagulants | B01A | Antithrombotic agents |
|  | Exclude: aspirin, clopidogrel, dipyridamole, prasugrel, selexipag, ticagrelor | |
| Antipsychotics | N05A | Antipsychotics |
| Psychotropics | N06A | Antidepressants |
|  | N04B | Dopaminergic agents |
|  | N03A | Antiepileptics |
